# Overturning in the subpolar North Atlantic: a review

**DOI:** 10.1098/rsta.2022.0191

**Published:** 2023-12-11

**Authors:** M. Susan Lozier

**Affiliations:** Georgia Institute of Technology, School of Earth and Atmospheric Sciences, Georgia Institute of Technology, Atlanta, GA, USA

**Keywords:** OSNAP, subpolar North Atlantic, meridional overturning circulation

## Abstract

The Overturning in the Subpolar North Atlantic Program (OSNAP) was initiated in the spring of 2010 through a collaborative effort involving the USA, the UK, Germany, the Netherlands and Canada. A key feature of OSNAP is a trans-basin observing system deployed in the summer of 2014 for the continuous measure of volume, heat and freshwater fluxes in the subpolar North Atlantic. This review focuses on advancements made possible by the collective OSNAP observations. Chief among those advancements is the quantification of the dominant role of the eastern subpolar North Atlantic in the production of dense waters that reside in the lower limb of the overturning: the Irminger and Iceland basins contributed approximately three times as much dense water compared with the Labrador Sea over the observational period. Other advancements include elucidation of the relationship between convective activity in the basin interior and boundary current anomalies; the spread of overflow waters in the subpolar region; the seasonality of the meridional volume, heat and freshwater fluxes; and the challenges involved in designing a simpler, less costly observing system. Collectively, OSNAP measurements are laying a framework on which to assess the overturning circulation's vulnerability to continued warming and freshening as climate change continues apace.

This article is part of a discussion meeting issue ‘Atlantic overturning: new observations and challenges’.

## 1. Introduction and background

The Atlantic meridional overturning circulation (AMOC), characterized by a northward flux of warm, saline upper-ocean waters and a compensating southward flux of cool, fresh deep waters, plays a fundamental role in establishing the mean climate state. To this point, the AMOC in the subtropical North Atlantic is largely responsible for the oceanic poleward heat flux at 26.5° N [1], an amount that accounts for 70% of the ocean's global contribution and 25% of the total poleward heat flux.

The AMOC is also considered responsible for the strong uptake of carbon at high latitudes: as northward-flowing surface waters cool they absorb additional CO_2_ that is carried to depth when deep waters form. This process makes the North Atlantic a strong sink for atmospheric CO_2_ [2], accounting for 41% of the annual mean global air–sea CO_2_ flux, with nearly half of that flux occurring north of 50° N.

Several decades ago, the study of the ocean's overturning was largely the purview of paleoceanographers who interpreted variability in deep ocean temperatures on millennial time scales in the context of what was then colloquially termed the ‘conveyor belt’ [3]. Alternate periods of global cooling and warming were attributed to changes in the ocean's overturning, brought about by the cessation or diminishment of deep-water production at high latitudes in the North Atlantic. However, studies in the 1990s indicated that changes in global atmospheric temperatures had occurred on the time scales of decades, or even years [4,5]. The proposed mechanism for the disruption was the ocean's overturning. With the publication of these studies, the distance between the paleoceanographer's world and the physical oceanographer's world collapsed. The possibility of abrupt climate change loomed in the near, not distant future.

As such, attention at the start of this century was sharply focused on the possibility of abrupt climate change caused by a collapse of the overturning circulation [6]. This worrisome scenario was based on the expectation that increasing carbon dioxide in the atmosphere would result in warmer surface waters at high latitudes. Those warmer waters would in turn stratify the upper water column to the extent that the formation of deep waters in the winter would cease, effectively shutting down the conveyor belt. This concern was coupled with another, namely that the oceanographic community had no direct measures of the meridional overturning circulation. This lack was a clear impediment to the understanding, monitoring and prediction of AMOC changes.

To meet this concern, a team of UK and US oceanographers designed and deployed an array of instruments across the North Atlantic basin at 26° N to provide the first continuous direct measure of the overturning. The Rapid Climate Change-Meridional Overturning Circulation and Heat Flux Array (abbreviated herein as the RAPID array), deployed in 2004, consists of moored instruments along the western and eastern boundaries of the basin and on either side of the Mid-Atlantic Ridge. This array complements a long-standing measure of flow through the Florida Straits [7] and is accompanied by a satellite measure of the directly wind-forced surface currents.

One year after deployment, the data were recovered and analysed to yield a time series of the overturning strength at that latitude. These results [8] challenged our understanding of AMOC's temporal variability. Just 2 years prior to this study, five synoptic surveys taken over the span of five decades were used to ascertain the long-term slowdown of the overturning [9]. In that study, as in past studies, the expectation was that the overturning varied slowly. Thus, it was assumed that a synoptic survey, which lasted weeks, would suffice to give more or less an annual measure of the overturning. The RAPID array results turned this expectation on its head by revealing exceptionally strong variability on times scales much shorter than a year: over the course of 1 year of continuous measurements the overturning strength at 26° N increased sixfold.

At the time of the RAPID array deployment, a continuous nature of the overturning was generally assumed. In other words, following the paradigm of a conveyor belt, oceanographers expected that overturning changes measured at one latitude would match the overturning changes measured at another. Thus, when the RAPID array was deployed in 2004, the general expectation was that it would measure *the* AMOC. However, shortly after this deployment, a modelling study suggested that AMOC fluctuations on interannual time scales are coherent only over limited meridional distances [10]. In particular, a break point in coherence at the subpolar/subtropical gyre boundary in the North Atlantic was evident in climate and ocean model output [10]. Following on the heels of that modelling study, another provided context for this lack of coherence [11]: modelled AMOC variability showed a gyre-scale response to variable wind forcing, yet a basin-scale response to buoyancy forcing. This study also suggested that buoyancy-forced AMOC changes, coherent throughout the basin, have larger amplitude in the subpolar North Atlantic and, as a result, are relatively less obscured by wind-forced signals there, making them potentially more observable.

At the same time as these modelling studies, the divide between the subtropical and subpolar regions was also becoming evident from Lagrangian observations in the subpolar North Atlantic. A study of PALACE floats released in the boundary currents of the Labrador Sea in the 1990s to track the pathways of newly formed Labrador Sea Water (LSW) [12] and a study of RAFOS floats in the early 2000s placed in the Deep Western Boundary Current (DWBC) at the exit of the Labrador Sea [13] to do the same, revealed a distinct discontinuity in the boundary current pathways at the Flemish Cap. A quantitative analysis of the RAFOS floats, as well as an accompanying model study, revealed that the dominant pathway for these deep waters to transit the subtropical ocean was in the interior, not along the western boundary [13]. Unknown at the time was whether the densest waters carried in the AMOC lower limb―those formed north of the Greenland-Scotland Ridge in the Nordic Seas and referred to as overflow waters―also followed interior routes or whether they were more likely to be advected along the DWBC.

These studies collectively motivated the international oceanographic community to launch a second AMOC observing system in the subpolar North Atlantic. Here, it was reasoned, would be the best chance to understand the coupling between buoyancy-forced deep-water formation and AMOC strength. Led by the USA in partnership with the UK, Germany, the Netherlands and Canada, and with contributions from France and China at various stages, planning for this observing system―Overturning in the Subpolar North Atlantic Program (OSNAP)―commenced in 2010 and the observing system was fully deployed in the summer of 2014.

The OSNAP array was designed to provide a continuous record of the overturning circulation and its associated fluxes of heat and freshwater in the subpolar North Atlantic [14]. The OSNAP was initiated with these five objectives: (i) to quantify the subpolar AMOC and its intra-seasonal to interannual variability via overturning metrics, including associated fluxes of heat and freshwater; (ii) to determine the pathways of overflow waters in the North Atlantic subpolar gyre to investigate the connectivity of the deep boundary current system; (iii) to relate AMOC variability to deep water mass variability and basin-scale wind forcing; (iv) to determine the nature and degree of the subpolar-subtropical AMOC connectivity; and (v) to determine from new OSNAP measurements the configuration of an optimally efficient long-term AMOC monitoring system in the North Atlantic subpolar gyre.

We are now more than a decade past the formulation of OSNAP and just under a decade from when it was first put in the water. The aim of this paper is to review the progress we have made on these objectives in the intervening decade. As such, I have structured this review around these five objectives. While there have been dozens of papers that have resulted from the individual arrays that constitute the OSNAP effort [15], this review focuses on the integrated measure of the meridional overturning circulation and the meridional fluxes of heat and freshwater that have been made possible by the entire array. The reader is referred to a previous study [14] for background on the observing system design and another [16] for the methodology used for the calculation of all OSNAP metrics. In the following section, I provide further background on the motivation for OSNAP. In §3, I provide an update on each of the five OSNAP objectives. A discussion of results is presented in §4 and a summary is provided in §5.

## 2. Major motivating questions at the outset of OSNAP

The motivation for OSNAP that I offered in the previous section focused on what we were learning about the subtropical AMOC from the RAPID array and about the subtropical/subpolar divide from modelling studies and Lagrangian observations. However, OSNAP was also motivated in large part by observations in the *subpolar* North Atlantic that did not square with our expectations in the early part of this century. Specifically, we expected at that time that downstream DWBC variability was a measure of AMOC variability [17] and that convection variability in the Labrador Sea was driving that AMOC variability. Observations showed otherwise. Specifically, measurements of the boundary currents east of the Grand Banks at 43° N during 1993 to 1995 and then again from 1999 to 2001 showed that transport in the LSW density range was remarkably steady despite the fact that LSW production was considerably weaker during the latter time period [17–20]. Similarly, a *strengthening* of the Deep Labrador Current at 53° N was recorded over the time period of a well-documented decrease in convection [21]. Finally, transport measurements of the DWBC equatorward of the Grand Banks appeared weaker when it advected a larger fraction of LSW [22].

One possible reason for the apparent disconnect between convection in the Labrador Sea and DWBC variability was that not all export pathways of these dense waters had been monitored in these studies. Though the DWBC had traditionally been considered the sole conduit for the lower limb of the AMOC, observational and modelling studies, as mentioned above, were revealing the importance of interior, as well as boundary, pathways [13,23–25]. In short, we could no longer be certain that DWBC variability was a reflection of AMOC variability. This uncertainty underscored the need for a trans-basin measure of the AMOC.

Secondly, a compilation of studies at the turn of the century [26–29] yielded a description of LSW production whereby the properties and transport variability within the DWBC were not a sole function of deep-water formation. Instead, boundary current transport, property gradients between the interior and the boundary current and the strength of the eddy field were suggested to play a role in setting the exit transport and properties of LSW. Thus, downstream LSW variability could not be assumed to reflect convective activity in the central Labrador basin. As such, the need to ascertain the linkage between convective variability in the basin interior and water mass variability in the boundary current was apparent.

Finally, it was suggested that the linkage between AMOC variability and deep-water formation could be impacted by wind-driven changes that drive overturning variability. Since the density field near the basin boundaries sets the overall shear of the basin-wide geostrophic circulation, wind-forced changes in that density field can modify AMOC strength [30]. In fact, AMOC changes on seasonal time scales have been linked to wind-forced Ekman pumping near the eastern boundary of the RAPID array [31] and similar processes involving Rossby wave transmission of Ekman pumping signals to the western boundary have been implicated on interannual to decadal time scales [32]. In short, it was recognized that AMOC variability at subpolar latitudes could also be affected by wind forcing.

Thus, while modelling studies had suggested a linkage between LSW formation and AMOC variability, a review during the OSNAP planning period [33] concluded that no conclusive observational evidence for a link between dense water formation in the Labrador Sea and AMOC variability had emerged to date. Thus, OSNAP was planned to provide sustained trans-basin measures of AMOC variability, contemporaneous with measures of dense water mass variability and basin-scale wind forcing in order to sort out this relationship.

## 3. OSNAP results

**Figure 1.**
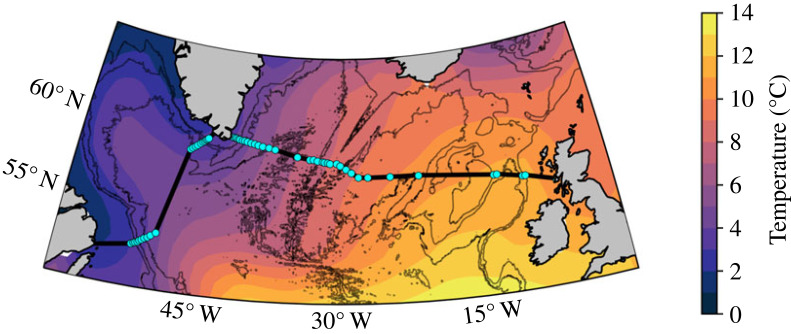
OSNAP sections in the subpolar North Atlantic. OSNAP West and OSNAP East are denoted with black lines and OSNAP mooring positions are indicated with cyan dots. Also shown is the climatological (1970–2020) sea surface temperature from EN4 [34,35]. Bathymetry is contoured at 1000, 2000 and 3000 m from GEBCO [36].

To aid the discussion of OSNAP results to date, I offer here a brief description of the observing system. OSNAP consists of two legs: one extending from the southern Labrador shelf to the southwestern tip of Greenland across the Labrador Sea and a second from the southeastern tip of Greenland to the Scottish shelf (figure 1). OSNAP was configured to capture the conversion of warm and salty North Atlantic waters in the upper ocean (on the eastern side of the basin, as seen in figure 1) into colder, fresher and dense waters. The sharp sea surface temperature contrast across the OSNAP array is attributed to strong heat loss along the cyclonic path of the wind-driven subpolar gyre: it is this heat loss that is largely responsible for deep water production at these latitudes.

OSNAP mooring arrays are located at the continental boundaries, as well as on both flanks of the Reykjanes Ridge. These arrays allow for the direct measure of temperature, salinity and velocity, while dynamic height moorings at key locations allow for the estimate of geostrophic flows. Argo float data, satellite altimetry and climatological property data are also used in the calculation of the OSNAP metrics. For the first 6 years of its operation, the observing system also included subsurface floats that were released and tracked in order to ascertain pathways of the overflow waters in the subpolar North Atlantic [37].

Because we are interested in the conversion of buoyant water moving northward (the upper AMOC limb) into denser waters moving southward (the AMOC lower limb), we use density coordinates for our calculation of MOC. (Note: For the purpose of this paper, I refer to ‘AMOC’ as the general circulation feature and reserve ‘MOC’ for its quantification.) Specifically, MOC is defined as the maximum of the overturning stream function (Sv) in density space. The OSNAP metrics also include the meridional heat transport (MHT) and meridional freshwater transport (MFT). All metrics are calculated as monthly means. Further information about the MOC, MHT and MFT calculations can be found in a previous study [16]. Finally, I will use the convention of describing waters within the AMOC in two categories―Upper North Atlantic Deep Water (UNADW) and Lower North Atlantic Deep Water (LNADW)―with the former produced within the subpolar basins and the latter denoting both overflow waters produced in the Nordic Seas and dense waters formed via entrainment as overflow waters spill into the North Atlantic over the Greenland-Scotland Ridge.

### (a) Quantify the subpolar Atlantic meridional overturning circulation and its intra-seasonal to interannual variability via overturning metrics, including associated fluxes of heat and freshwater

Statistics on the MOC, MHT and MFT from each of the three 2-year ‘batches’ of OSNAP data have previously been reported [38–40]. A brief summary of the statistics on the 6-year time series is offered below.

#### (i) Mean MOC, MHT and MFT

**Figure 2.**
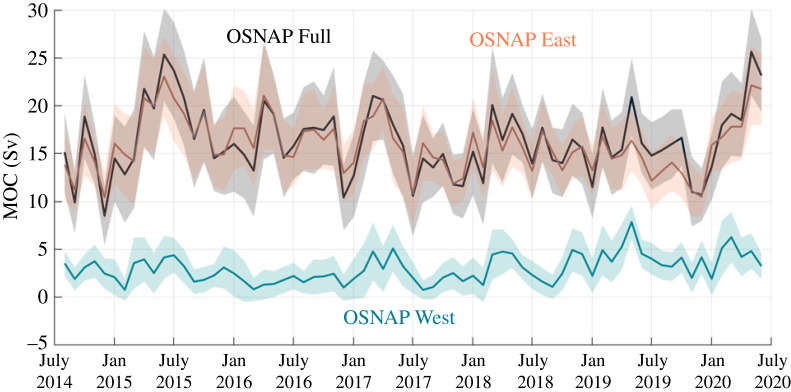
OSNAP MOC time series. Monthly MOC time series across the full OSNAP array (black), OSNAP East (orange) and OSNAP West (cyan). Shading indicates the uncertainty estimated from a Monte Carlo analysis. Figure is reproduced from Fu *et al.* [40].

The major result to date from the integrated OSNAP system―that overturning across OSNAP East far surpasses that across OSNAP West―was revealed by an examination of the first 2 years of observations (2014–2016) [38], confirmed by the extension of the time series to 2018 [39] and evident in the 6-year time series shown in figure 2 [40]. For the 6-year time series, the mean MOC (± standard error) across OSNAP West is 3.0 ± 0.5 Sv, compared with 16.3 ± 0.6 Sv across OSNAP East and 16.7 ± 0.6 for the total OSNAP [40].

Because of the strong dependence of MHT on the overturning circulation [25], the mean MHT is also dominated by OSNAP East. MHT across this line contributes 0.42 ± 0.01 PW of the total (0.50 ± 0.01 PW), with OSNAP West contributing only 0.08 ± 0.01 PW. However, OSNAP West plays a relatively large role in the southward flux of freshwater, contributing −0.17 ± 0.01 Sv to the total MFT of −0.36 ± 0.01 Sv, while OSNAP East contributes a comparable −0.18 ± 0.01 Sv. This stronger showing for OSNAP West's MFT (compared with its MHT) is attributed to the strong salinity contrast on opposing sides of the basin in the upper water column: relatively salty waters imported from the Irminger Sea move northward along the eastern boundary of the basin, while much fresher waters, augmented by Arctic waters flowing southward via the Davis Straits, are advected along the Labrador shelf/slope.

#### (ii) MOC, MHT and MFT variability

Because the modelling studies referenced above led to an expectation that MOC variability at high latitudes would be dominated by lower frequencies than those at RAPID, the strength of the month-to-month variability in the OSNAP record came somewhat as a surprise. As it turns out, its monthly range of 10–25 Sv over the 6-year time series [40] is comparable with that at RAPID [41]. OSNAP East dominates the variability in MOC, accounting for 83% of the total variance. Likewise, MHT variability is dominated by OSNAP East, accounting for 91% of the total variance. The story is a bit different for MFT since, as in the mean, OSNAP East and West have comparable contributions: OSNAP West can explain 42% of the total MFT variance, while OSNAP East explains 46%.

#### (iii) MOC, MHT and MFT seasonality

The first determination of the MOC, MHT and MFT seasonal variability [40] has revealed that 40%, 21% and 55% of the total MOC, MHT and MFT variability, respectively, can be explained by seasonal variability. In brief, MOC seasonality is driven by the export of newly-formed deep water across OSNAP East and West several months after formation and by seasonally-varying Ekman transport that actually damps the seasonality imposed by the buoyancy signal. The relatively weak contribution of seasonality to the overall MHT variability is due to the strong southward Ekman transport across OSNAP East in the winter, which reduces the total northward MHT. On the other hand, the MFT has the most pronounced seasonal signal of these three metrics owing in large part to the seasonality in the export of fresh coastal waters of Arctic origin along the shelf and slope of the Labrador Sea [40].

### (b) Determine the pathways of overflow waters in the North Atlantic subpolar gyre to investigate the connectivity of the deep boundary current system

As mentioned earlier, previous observational work had elucidated the downstream pathways for the LSW, but as pointed out in a recent review [42], the pathways of the Nordic Seas overflow waters had not been directly observed prior to OSNAP, only simulated from numerical output [43]. Major results of the OSNAP float program are reviewed here.

#### (i) OSNAP float program

OSNAP was purposely placed downstream of the entry of the Iceland-Scotland Overflow Water (ISOW) and Denmark Strait Overflow Water (DSOW) into the North Atlantic [14]. Because this placement is sufficiently removed from the sills of the Greenland-Scotland Ridge, these water masses have reached their neutrally-buoyant depths by the time they move through the OSNAP arrays. As such, isobaric RAFOS floats were sufficient to track their downstream pathways. Daily position fixes for each float were made possible from an array of moored sound sources in the subpolar North Atlantic [37].

In total, 135 RAFOS floats were deployed from 2014 to 2018 primarily at three OSNAP array sites: on the eastern and western flanks of the Reykjanes Ridge and to the east of the Greenland coast. Floats released in the eastern Reykjanes Ridge array tracked ISOW, those released in the western Reykjanes Ridge array tracked Northeast Atlantic Deep Water (NEADW) and those released off the east coast of Greenland tracked both NEADW and DSOW. (Note: NEADW shares the same density range with ISOW, but because its origin is not directly, or even easily, traced to Nordic waters flowing over the same sills as ISOW, it has been common to use this generic label [20,44,45].) For convenience, the term ‘overflow water’ here is used to refer to the collection of ISOW, DSOW and NEADW. These are also the waters referred to as LNADW.

#### (ii) Observed overflow water spreading pathways

**Figure 3.**
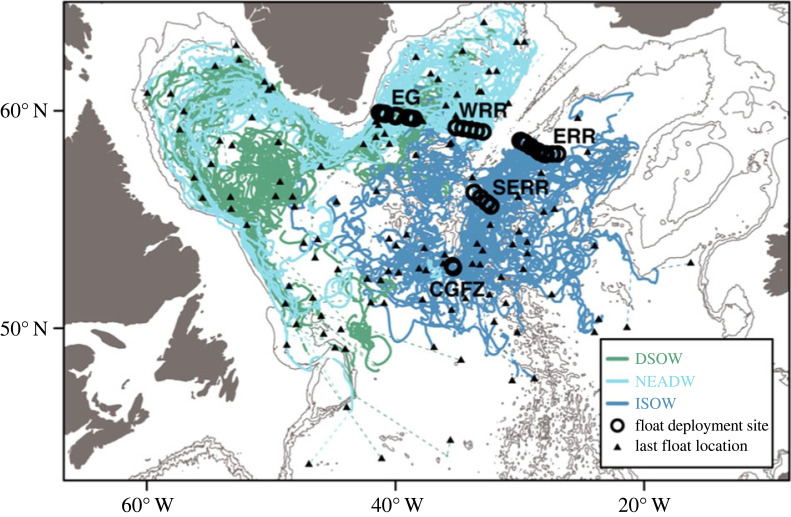
Overview of all OSNAP float trajectories. The five float deployment sites are: EG, east of Greenland; WRR, west Reykjanes Ridge; ERR, east Reykjanes Ridge; SERR, southeast Reykjanes Ridge and CGFZ, Charlie-Gibbs Fracture Zone. Trajectories are colour coded to show floats embedded at launch in ISOW at ERR, SERR and CGFZ (dark blue); NEADW at WRR and EG (light blue); and DSOW at EG (dark green). (Note: in this review, the focus is on the three main launch sites: EG, WRR and ERR.) Circles mark launch locations, triangles mark surface locations at floats' mission end, and dashed lines connect trajectory segments where acoustic tracking was not possible. The bathymetry is contoured at 1000, 2000 and 3000 m. Details on the OSNAP float program can be found in a prior study [37]. Figure is adapted from Lozier *et al.* [46].

As detailed in a recent paper [46], a main result from the OSNAP float program, as evidenced by the compilation of all 2-year trajectories (figure 3), is that though all floats were purposely released in deep boundary currents of the subpolar North Atlantic, they ‘fill’ the interiors of the Iceland, Irminger and Labrador basins during their lifetimes. The spread of these overflow waters out of the boundary currents is reminiscent of that previously observed for LSW as it exits the Labrador basin [13]. A second main result is that floats exit the Irminger basin much more quickly than they exit the Iceland basin. Floats tracking DSOW and NEADW out of the Irminger basin were advected into and around the Labrador basin over the course of 2 years, while those tracking ISOW, launched in the Iceland basin, were much less likely to leave that basin over the 2 years of the floats' lifetime. The use of Markov chain simulations ([47] and references therein) allowed for a quantification of these differences: over 90% of the particles launched in the Irminger basin, whether tracking DSOW or NEADW, leave that basin after 2 years, while only 50% of the particles launched in the Iceland basin, and tracking ISOW, leave that basin.

The third main result from the observational float program, related to the second, is the striking difference in spreading pathways between the Irminger and Iceland basins. Just one dominant route was evident for floats exiting the Irminger basin, namely the swift boundary current along east Greenland. Almost all floats were swept into this boundary current, rounded Erik Ridge and entered the Labrador Sea via this route. By contrast, floats that escaped the Iceland basin did so by multiple pathways: across the Reykjanes Ridge via gaps north of the Charlie Gibbs Fracture Zone, westward through the Charlie Gibbs Fracture Zone and southward to the West European Basin [25,48–50]. Finally, as detailed in Zou *et al.* [49], there is no evidence for a robust ISOW pathway into the Irminger Sea from the Charlie Gibbs Fracture Zone, a result recently confirmed from a calculation of the mean velocity field at the depth of the overflow waters using OSNAP float data [51]. This analysis showed no evidence for a deep boundary current that wraps around the Reykjanes Ridge connecting the Iceland and Irminger basins, a pathway that has traditionally been considered the main route for ISOW to leave the Iceland basin.

#### (iii) Modelled long-term fate of overflow water pathways

**Figure 4.**
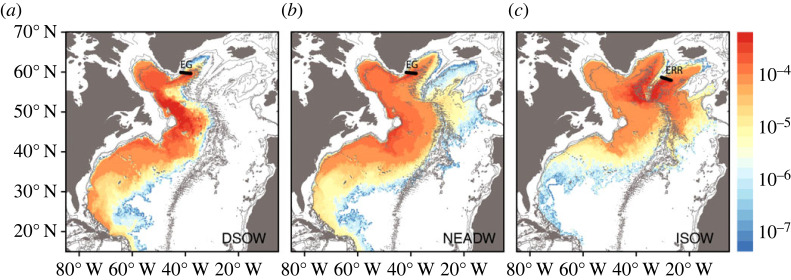
Modelled long-term fate of overflow waters. Cumulative 10-year probability distributions of particles launched in DSOW from EG (*a*), NEADW from EG (*b*) and in ISOW from ERR (*c*) using output from HYCOM, an eddy-resolving ocean general circulation model. The HYCOM version used for this study has previously been used to investigate water masses in the deep North Atlantic and previously validated with observations [50,52]. To produce these maps, 5880 particles were launched in DSOW from EG; 5940 particles were launched in NEADW from ER; and 6600 were launched in ISOW from ER. The probability distribution is shown as the percentage of particle positions such that the total value of all grid cells add up to 100%. Launch locations are shown as black lines. Bathymetry is contoured at 1000, 2000 and 3000 m. Figure is adapted from Lozier *et al.* [46].

The observational OSNAP float program was complemented by a modelling study that extended the integration time for the pathways [46]. Using output from two ocean circulation models, the observational launch strategy was mimicked, meaning that numerical particles were launched from the same locales as the RAFOS floats and they sampled the same water masses, namely DSOW, NEADW and ISOW (figure 4). (Note: Since results from the two models are similar, figure 4 shows results from one model only. The reader is referred to Lozier *et al.* [46] for the description of both models.) Of course, the advantage of using numerical output is that thousands of numerical trajectories were simulated, each integrated for 10 years from its launch.

This numerical study confirmed the main observational results that pertained to the spread of the overflow waters in the subpolar North Atlantic and revealed stark differences in the spread of these water masses into the subtropical North Atlantic. While DSOW and NEADW primarily reside in the western North Atlantic in their equatorward transit, ISOW shows a strong preference for the eastern North Atlantic (i.e. east of the Mid-Atlantic Ridge in the West European Basin), a result consistent with previous studies [25,48,50]. Interestingly, though the OSNAP float program was designed to understand the differences in the spreading pathways for ISOW and DSOW, the observational and modelling studies revealed that the spreading pathways are more differentiated by their basin of origin than they are by their water mass characteristics. In other words, DSOW and NEADW share very similar spreading pathways despite differences in density for the simple reason that they are both entrained into the deep extension of the cyclonic boundary current in the subpolar North Atlantic. By contrast, ISOW, at the same density as NEADW, spreads more slowly along multiple pathways out of the Iceland Basin. It is surmised that these multiple pathways stem in large part from the influence of the overlying energetic North Atlantic Current in the region of the Charlie Gibbs Fracture Zone [48,49,53].

### (c) Relate Atlantic meridional overturning circulation variability to deep water mass variability and basin-scale wind forcing

The dominance of OSNAP East overturning raised two critical questions. The first related to the source of waters exiting the lower AMOC limb across OSNAP East. Namely, could buoyancy-driven surface transformation account for that volume of water? The second question was a simple one: Why was the overturning across OSNAP West so small compared with expectations and with modelling estimates? I take each of these in turn below.

#### (i) Link between mean surface-forced transformation and the MOC in OSNAP East

**Figure 5.**
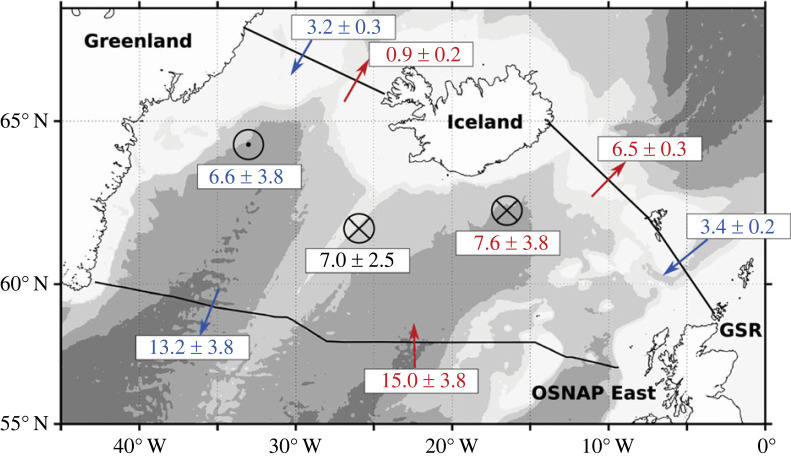
Accounting for dense waters in the eastern subpolar North Atlantic. Volume budget of the upper (red) and lower (blue) AMOC layers between the Greenland-Scotland Ridge and OSNAP East for the OSNAP period of 2014–2016. Circle with a central dot indicates the volume of water (Sv) received in the lower layer from the upper layer, estimated from the volume budget of the lower layer; circles with crosses indicate export of water from the upper layer to the lower layer, estimated from the volume budget of the upper layer and from the averaged transformation across *σ*_MOC_ (black, unit Sv). Bathymetry is shaded for 500, 1000, 2000 and 3000 m. Note that the red and blue arrows across OSNAP East schematically represent a number of significant currents that compose the upper and lower limbs and that the blue arrows across the Greenland-Scotland Ridge denote overflow waters. Reproduced from Petit *et al.* [54].

A volume budget for the area bounded to the south by OSNAP East and to the north by the Greenland-Scotland Ridge (figure 5) was constructed using OSNAP East measurements, atmospheric reanalyses, and the time series of volume transport across the Greenland-Scotland Ridge provided by the AtlantOS consortium [55]. The budget was calculated for two layers separated by the isopycnal that maximizes the cumulative northward transport in the MOC upper limb, referred to as *σ*_MOC_ [38]. This budget analysis showed that over the first 2-year OSNAP period (2014–2016), the mean volume of water exiting southward across OSNAP East in the MOC lower limb, after subtracting for the volume of overflow waters (6.6 ± 3.8 Sv), could be accounted for by the mean volume of water produced at the surface via buoyancy loss (7.0 ± 2.5 Sv). Similarly, this transformation volume is a nice match to the upper limb volume convergence (7.6 ± 3.8 Sv), which is the difference in transport between the northward transports across OSNAP East and the Greenland-Scotland Ridge. A repeat of this analysis for the Labrador basin revealed a similar result: the volume of surface buoyancy-driven water mass transformation (1.5 ± 0.7 Sv) can account for the mean overturning in that basin (2.1 ± 0.3 Sv) over the 2-year OSNAP period. Similar analyses are planned for the longer OSNAP time series to see if these results hold, however we note here that a similar analysis over the domain from 45° N to the Greenland-Scotland Ridge using indirect measures of the velocity field (i.e. geostrophic velocities referenced with altimetry-derived surface velocities) over approximately two decades also yield an approximate match between mean surface transformation and the mean overturning [56]. Thus, the large OSNAP East overturning measure over these first two OSNAP years can be accounted for in the mean by summing (i) the collective overflow water transport and (ii) the production of dense waters via surface transformation in the eastern subpolar North Atlantic. Complementing this analysis, a recent study [57] has revealed the important role of mixing in redistributing the volume in the density classes that reside within the AMOC lower limb.

#### (ii) Density compensation across OSNAP West

**Figure 6.**
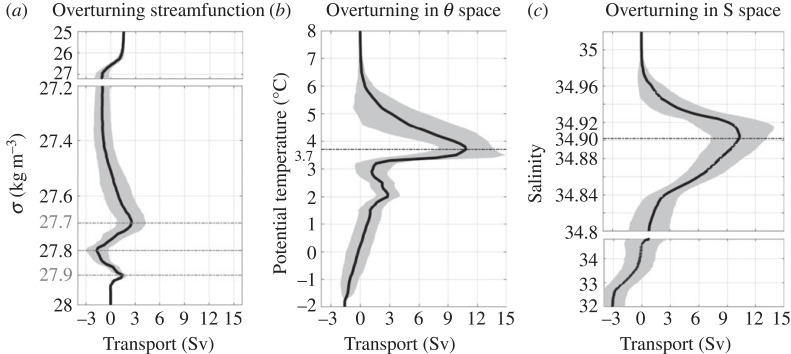
(*a*) The mean overturning streamfunction from August 2014 to April 2016 along OSNAP West, with its monthly standard shaded in grey. (*b*) The overturning streamfunction with respect to temperature space, with a maximum reached at 3.7°C. (*c*) The overturning streamfunction with respect to salinity space, with a maximum reached at 34.90. Adapted from Zou *et al.* [61].

Strong cooling during the first two winters following OSNAP's deployment raised expectations that the first direct measure of OSNAP West overturning would be robust. Contrary to that expectation, the first 2-year time series revealed a weak overturning, at least relative to OSNAP East, as mentioned above, and relative to model estimates [58]. Though mixed layers were recorded to depths of 1500–2000 m in the Labrador Sea [59,60] at the end of those winters, the volume of light water transformed into denser water (i.e. denser than *σ*_MOC_ for OSNAP West) was only 2.1 ± 0.3 Sv (figure 6*a*).

To reconcile the simultaneous occurrences of strong cooling and weakening overturning, the overturning analysis was expanded to include overturning in temperature and salinity space [61]. In other words, the volume of warm water converted to cold water across OSNAP West (figure 6*b*) and the volume of salty water converted to fresh water (figure 6*c*) across the same line were separately computed. Interestingly, the overturning in both temperature (13.9 ± 3.0 Sv) and salinity space (11.4 ± 2.8 Sv) exceeds that in density space by a factor of approximately 6. This analysis demonstrates that overturning in density space was limited in the Labrador basin due to density compensation: warm salty waters of Irminger Sea origin were both cooled and freshened in their transit through the Labrador basin. The densification due to cooling was nearly offset by the de-densification due to freshening. A follow-on study using OSNAP observations in the context of an idealized three-layer model [62] finds that density compensation of the boundary current waters can be explained by strong heat loss to the atmosphere and the simultaneous exchange of boundary current waters with the much fresher shelf waters rimming the Labrador basin.

#### (iii) Impact of wind-forcing for OSNAP East and West

While there is a nice match between mean surface transformation and the mean export across OSNAP East of waters formed in the eastern subpolar region, there is no such match on monthly time scales [54]. Instead, there is large variability in the storage of these waters (calculated as the difference in the production and export) from month to month. Moving forward, we plan an analysis of the mechanisms that drive export variability on intra-seasonal and interannual time scales. We surmise that local wind forcing plays a large part in the release of dense waters from these subpolar basins. Likely the impact of wind forcing―in addition to that provided by Ekman dynamics―is similar to that at RAPID [63], where isopycnal heaving can explain a large fraction of the intra-seasonal and interannual variability.

### (d) Determine the nature and degree of the subpolar–subtropical Atlantic meridional overturning circulation connectivity

For the common overlapping observational period of 2014–2020, the MOC at RAPID and at OSNAP are comparable in the mean, 16.8 ± 0.5 Sv for the former [64] and 16.7 ± 0.6 Sv for the latter [40]. The mean MFTs and MHTs are not similarly matched due to the significant exchange of heat and freshwater between the two lines [65]. No relationship, however, is found between the RAPID and OSNAP MOC temporal variability over the 6 years of overlapping data in hand. The same holds true for MHT and MFT variability. This result comes as no surprise given the short overlapping period relative to the strong intra-seasonal and interannual variability exhibited at both locations. Our updated understanding of the Lagrangian pathways for both UNADW and LNADW also helps explain this disconnect at the subtropical–subpolar boundary, as does what we know about tracer ages in the North Atlantic [66]. While some UNADW that leaves the Labrador basin is exported to the subtropical gyre, even more recirculates in the subpolar basin [13]. This differentiation of fate leads to a sizeable range of ages of UNADW in the subtropics [43]. The same is expected for LNADW, especially so for ISOW because of the multiple pathways it takes to reach the subtropics. However, regardless of how anomalies are propagated from the subpolar region to the subtropics―that is whether they propagate downstream via boundary waves or advective processes―it is generally understood that coherence is unlikely on interannual time scales [67,68].

While we cannot find any correlation between the OSNAP and RAPID time series to date, I will note here that LNADW transport exhibits greater variability compared with that for UNADW at the RAPID array [69]. This result is interesting since the opposite is true across OSNAP East [54], suggesting that the downstream variability observed at RAPID is *not* the result of upstream source water variability. More on this point will be discussed in the next section.

### (e) Determine from new OSNAP measurements the configuration of an optimally efficient long-term AMOC monitoring system in the North Atlantic subpolar gyre

**Figure 7.**
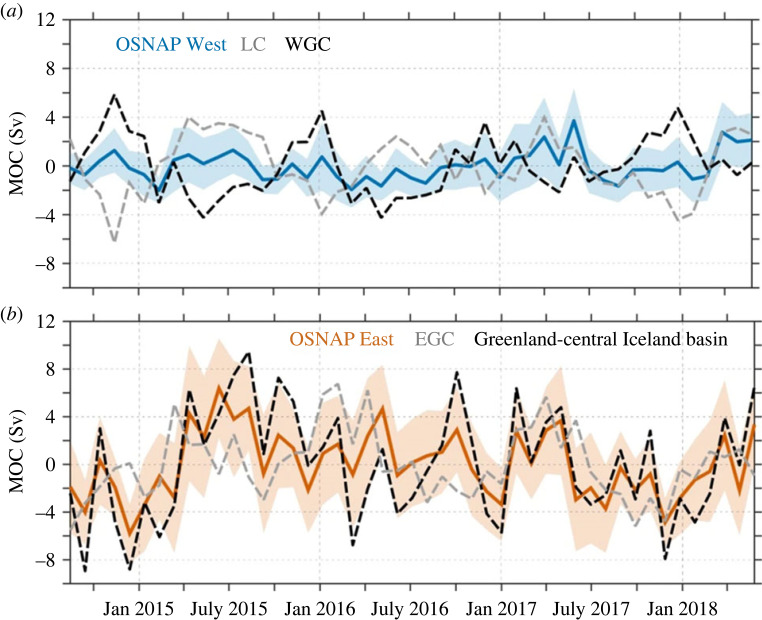
Components of overturning variability. (*a*) OSNAP West MOC variability (blue; shading indicates uncertainty); MOC variability arising from time-varying density and velocity anomalies in the Labrador Current (LC; light grey) computed with time-mean velocities/densities at the West Greenland Current (WGC) boundary; and MOC variability arising from density and velocity anomalies in the WGC (black) computed with time-mean velocities/densities at the LC boundary. (*b*) OSNAP East MOC variability (red; shading indicates uncertainty); MOC variability arising from time-varying density and velocity in the region between Greenland and mid-Iceland basin (black) computed with time mean velocities/densities at the eastern boundary; and MOC variability arising from density and velocity anomalies in the East Greenland Current (EGC; light grey) computed with time-mean velocities/densities everywhere else. For the reconstruction based on the time-varying data at the western boundary (light grey line), the MOC is defined as the minimum of the stream function integrated from the bottom to the sea surface in density space (sign has been changed to aid a comparison). Reproduced from Li *et al.* [39].

Efforts are currently underway on this objective. Our long-term goal is to transition to a more efficient, i.e. less costly, observing system after 10 years of observations with the current observing system. The past 6 years of observations have led to the following insights and principles that are guiding our work toward this long-term objective:

1. The separate lines of OSNAP East and West have been invaluable to our understanding of where the majority of deep waters are formed in the subpolar North Atlantic. The ability to differentiate the amount of overturning in the western versus eastern subpolar North Atlantic and, importantly, the mechanisms responsible for variability in both regions is critical for our continuing assessment of how the overturning in this region will be affected by the expected warming and freshening of the surface waters in the years and decades ahead.
2. As earlier reported [39], the variability at either OSNAP West boundary array can account for less than 10% of the total MOC variability in that basin (figure 7*a*). An analysis of those boundary anomalies reveals that anomalies exported in the boundary array off of Labrador are strongly correlated with those imported on the opposite side, meaning that the anomalies are advected around the basin's rim. Thus, the measurement of density anomalies at any one boundary is not relevant to a measure of transformation in that basin. Similarly, at OSNAP East, boundary current variability off eastern Greenland can also explain only about 10% of the total MOC variability (figure 7*b*) [39]. A meaningful fraction of the MOC lower limb variability (75%) is captured only by including a broad swath that stretches from the eastern boundary to the Iceland basin. These results preclude a simple approach to the design of a less costly observing system as it appears that no boundary array is expendable. Thus, current efforts are focused on ‘thinning’ the arrays, all the while an exploration of alternate means to assess the subpolar MOC, MHT and MFT continues. As for the latter, it is hoped that continued ground truthing of ocean models with OSNAP data will lead to long-term monitoring that employs a combination of select *in situ* and satellite observations with numerical models.
3. An optimization of the array should account for MHT and MFT, as well as MOC. While OSNAP East dominates the MOC and MHT measures, OSNAP West plays a large―and at times dominant―role in the total MFT. While the prime focus of OSNAP was initially on the MOC, the continued measurement of the heat advected into the Nordic Seas and the freshwater advected from the Davis Strait, out of the Nordic Seas, and from Greenland glacial melt are important as we continue to understand the complex feedback mechanisms at play between the dynamics and thermodynamics of the Arctic region and subpolar North Atlantic. Thus, the next phase of the OSNAP observing system will likely place a strong emphasis on MHT and MFT.

## 4. Discussion of results

In this section, I first discuss the OSNAP results to date in the context of the main questions (§2) that motivated its deployment in 2014. I conclude with a discussion of how the OSNAP float program results aid our understanding of AMOC variability.

### (a) Sorting out the linkage between convective activity and Atlantic meridional overturning circulation variability

At the initiation of OSNAP, a question loomed as to whether or not boundary current variability reflected AMOC variability in the subpolar North Atlantic. We can now state with confidence that a measure of variability at any one subpolar boundary is insufficient to yield the total AMOC variability [39], nor can any one boundary in OSNAP East or OSNAP West capture a significant fraction of MOC variability across their respective sections. In large part, this insufficiency can be attributed to the fact that the swift cyclonic boundary current rimming the sub-basins of the subpolar North Atlantic is advecting anomalies formed in one part of the basin to other parts of the basin or from one basin to the next, as demonstrated in a recent modelling study [70]. In this regard, the subpolar gyre is remarkably different from the subtropical gyre in that there is strong variability at a number of boundaries that contribute to the total variability.

There was also at the start of the OSNAP a general expectation that convective activity in the central Labrador basin drove downstream AMOC variability. We can now say definitively that convective activity in the Labrador Sea does not drive downstream AMOC variability over the observational period. We have established that convective activity in the eastern subpolar North Atlantic produces far more dense water in the lower limb than that produced in the Labrador basin. The OSNAP has also established that there is only a weak linkage between density anomalies in the Labrador basin interior and those at its boundaries [39]. A recent modelling study [71] focused on the attribution of AMOC variability at 26° N found that anomalies in the boundary of the Labrador Sea played a far larger role in the downstream variability than those found in the basin interior, prompting the authors to state that ‘the causal connection between water mass transformation in the Labrador Sea and the subtropical AMOC is complex'.

That complexity, however, is reduced through spatial and temporal integration. We can now state unambiguously that over the observational period we can largely account for the amount of water exported out of the subpolar gyre by the sum of the overflow waters and those produced via surface transformation in the eastern subpolar North Atlantic. The waters produced in the Labrador Sea add to the sum, but they pale in comparison with the other two sources. In large part, we have learned what we might have guessed at the start, namely that the volume of waters that are exported out of the subpolar North Atlantic are the result of the densification of surface waters across a broad swath of the subpolar North Atlantic and Nordic Seas. A measure of the surface transformation over a particular subregion is inadequate for a complete accounting of deep water formation and a measure of transport at any one boundary is inadequate for a complete accounting of the export out of the subpolar North Atlantic. Even the boundary at the exit of the Labrador Sea misses the ISOW that spreads southward from the Iceland basin in its transit to the subtropical region. Furthermore, a portion of the UNADW that courses through the boundary array at 53° N recirculates in the subpolar basin. Thus, a trans-basin measure of the deep waters has been indispensable to our efforts to make a definitive linkage between deep water formation and the AMOC.

That certainty does not yet extend to understanding the link between deep water formation *variability* and MOC *variability*. We are hampered in this regard by a time series of just 6 years. While we have ascertained that the seasonal MOC variability is driven by a combination of convective activity and Ekman flow, we have yet to answer the degree to which buoyancy and wind forcing, whether remote or local, control MOC variability on interannual time scales. While the longer time series will aid our efforts in this regard, there is little doubt that our understanding of the MOC variability will rely on an integrated measure of that variability. In other words, there are (at least not yet) no obvious proxies for the MOC variability or, for that matter, there are no obvious proxies for variability in deep water formation. In fact, over the OSNAP period we have learned that proxies for overturning and those for deep water formation can lead us astray [72]. In hindsight, we now understand the simple fact that to capture the mean and variability of the formation and export of deep waters across the subpolar North Atlantic, we need integrated observations for both. We do not see any short-cuts at this point. At the risk of stating the obvious, AMOC is an integrative measure and to understand that integrated measure, we need a comprehensive observing system.

### (b) Implications of Lagrangian pathways for our understanding of the mean Atlantic meridional overturning circulation and its variability

We are decades away from the expectation that all water carried in the deep AMOC limb is channelled within deep boundary currents. Still, while we had observational evidence that this was indeed the case for UNADW, the OSNAP float program has provided evidence that LNADW also takes multiple routes in its equatorward transit. However, while most UNADW in its southward transit is advected within the western North Atlantic, either via the boundary current or along interior pathways, such is not the case for LNADW. In the case of ISOW, a significant portion of these waters [46,48] arrives in the subtropics having never transited through the Labrador basin or even in the western basin more broadly. Thus, a full accounting of the mean equatorward transport of LNADW requires a trans-basin measure.

A pressing question for a number of years has been the extent to which downstream MOC variability can be linked to variability in deep water formation at the source sites. While the OSNAP section has produced a match between the mean production of dense waters in the subpolar North Atlantic and their mean export, the Lagrangian element of the OSNAP has given us insight into this question. Specifically, an analysis of the OSNAP floats has confirmed what we have come to understand for UNADW, namely that the deep waters (UNADW or overflow waters) that are carried equatorward have a large range in age by the time they reach the subtropics. This age distribution is the result of (i) local recirculations in the sub-basins of the subpolar North Atlantic, causing entrainment into and detrainment from the boundary current; (ii) different routes for the waters to reach the subtropics; and (iii) mixing along those routes. Transit times for overflow water to reach the subtropics [43,46] are of the order of decades, not years, with the age range also measured in decades. Thus, these results cast strong doubt on an expectation that an anomalous production in deep water formation in one year can be traced downstream. Instead, we would expect that coherence in water mass anomalies would be achieved only on decadal- to multidecadal scales. The limiting factor here is essentially the residence time scale of the overflow waters within the subpolar basin [43,66,73].

## 5. Summary

Since inception, the integrative measure of AMOC across the OSNAP lines has revealed the dominance of overturning in the eastern subpolar North Atlantic―across a section stretching from Greenland to Scotland―rather than overturning across the Labrador Sea. This dominance is expressed in the mean and variability of this metric. Though this partitioning was initially at odds with that found in many model estimates, recently published model results now show this same partitioning [70,74].

Overturning in the Irminger and Iceland basins can largely be accounted for in the mean by the surface transformation of light waters in the upper AMOC limb to dense waters in the lower limb. However, the storage of newly formed deep waters shows sizeable variability from month to month. An investigation of the mechanisms governing the release of these waters on monthly and interannual time scales is planned with the next installment of the OSNAP time series.

Though the first years of the OSNAP were marked by the production of a large volume of cold and fresh waters in the Labrador Sea, the production of dense waters was not commensurate. Instead, density compensation―whereby the densification from cooling was largely offset by freshening―resulted in a relatively weak overturning. The 6-year OSNAP record suggests that this density compensation was not unique to the first 2 years of the program. An idealized modelling study suggests that the density-compensated overturning can be explained by a combined effect of the atmospheric cooling of the Irminger Water carried into the Labrador Sea boundary current and mixing of the Irminger Water with the fresh shelf waters off western Greenland and the Labrador coast. A major question in the years ahead concerns the degree to which ice/glacial melt will impact overturning in the subpolar North Atlantic. Though a recent study concluded that Greenland meltwater has not yet had an impact on overturning [75], at some point this freshening is likely to completely counter wintertime cooling such that dense water production in this basin is curtailed.

The OSNAP MHT is largely controlled by overturning dynamics. As such, the mean and variability of MHT is dominated by OSNAP East. This is not the case for MFT, where OSNAP West plays an outsized role for its size in terms of setting the mean and variability. The fresh waters along the western boundary of the Labrador basin strongly impact the total OSNAP MFT. As such, we have worked to improve our measure of the flow field and properties along the Labrador slope/shelf, particularly in light of the fact that we expect these waters to rapidly change their properties as ice melt accelerates.

The OSNAP float program has revealed remarkably different spreading patterns for DSOW and ISOW in the subpolar North Atlantic. ISOW spreads southward along multiple pathways, including those to the east of the mid-Atlantic Ridge, while DSOW spreads primarily along pathways that lead into the Labrador basin and then southward to the subtropics along pathways to the west of the mid-Atlantic Ridge. The Lagrangian view of these overflow waters aids our interpretation of the time scale for overturning anomalies to move downstream.

A redesign of the OSNAP observing system in order to reduce its complexity and cost has been hampered by the realization of the complexity of the overturing dynamics. We now understand that anomalies in a single boundary current do not capture a meaningful amount of AMOC variability, particularly in the Labrador Sea. The link between convection in the interior and boundary current anomalies is not straightforward because anomalies found in the boundary current can be imported from upstream, created due to exchange with the interior, and formed in the boundary current itself. In large part, we now fully understand that the measure of an integrative metric requires an integrated measuring system. This statement seems obvious now, but somehow this was not the case 10 years ago.

Though we have learned a considerable amount since the initiation of the OSNAP, key questions remain. Chief among those is whether or not the observational OSNAP period is representative of MOC, MHT and MOC variability on longer time scales. Though, as mentioned above, some modelling studies now show the same OSNAP East/West partitioning in the mean, a recent modelling study suggests that deep water formation in the Labrador Sea plays a dominant role on multidecadal time scales [74]. A second open question concerns the considerable spread among climate models in the AMOC response to a warming climate [76]. Whether this divergence results from differences in model resolution, representation of properties and transport of the overflow waters, mixing parameterizations, input of melt water from Greenland and the Arctic and/or a number of other processes remains an active area of research. Finally, a critically important question that motivated in large part the initiation of OSNAP concerns how changes in the AMOC will impact the uptake of anthropogenic CO_2_ [77,78] in the decades ahead.

## Data Availability

This article has no additional data.
